# IQOS: examination of Philip Morris International’s claim of reduced exposure

**DOI:** 10.1136/tobaccocontrol-2018-054321

**Published:** 2018-08-29

**Authors:** Gideon St.Helen, Peyton Jacob III, Natalie Nardone, Neal L Benowitz

**Affiliations:** 1 Division of Clinical Pharmacology, Department of Medicine, University of California San Francisco, San Francisco, California, USA; 2 Center for Tobacco Control Research and Education, University of California, San Francisco, California, USA; 3 Department of Bioengineering and Therapeutic Sciences, University of California San Francisco, San Francisco, California, USA

**Keywords:** electronic nicotine delivery devices, harm reduction, non-cigarette tobacco products, smoking caused disease, tobacco industry

## Abstract

**Background:**

New electronic heated tobacco products are being introduced in the global market and are gaining popularity. In 2016, Philip Morris International, Inc. (PMI) submitted a modified risk tobacco product (MRTP) application to the Food and Drug Administration (FDA) to market IQOS in the USA with claims of reduced exposure and reduced risk.

**Methods:**

We examined PMI’s MRTP application, specifically sections on aerosol chemistry and human exposure assessment, to assess the validity of PMI’s claims of reduced exposure and risk.

**Findings:**

PMI reported levels for only 40 of 93 harmful and potentially harmful constituents (HPHCs) on FDA’s HPHC list in IQOS mainstream aerosol. All substances in PMI’s list of 58 constituents (PMI-58) were lower in IQOS emissions compared with mainstream smoke of 3R4F reference cigarettes. However, levels of 56 other constituents, which are not included in the PMI-58 list or FDA’s list of HPHCs, were higher in IQOS emissions; 22 were >200% higher and seven were >1000% higher than in 3R4F reference cigarette smoke. PMI’s studies also show significantly lower systemic exposure to some HPHCs from use of IQOS compared with smoking combustible cigarettes.

**Conclusion:**

PMI’s data appear to support PMI’s claim that IQOS reduces exposure to HPHCs. However, PMI’s data also show significantly higher levels of several substances that are not recognised as HPHCs by the FDA in IQOS emissions compared with combustible cigarette smoke. The impact of these substances on the overall toxicity or harm of IQOS is not known.

## Introduction

Many alternative tobacco products have entered the USA market in the last three decades. These include electronic cigarettes that heat a nicotine solution^1^ as well as products that heat tobacco without combustion called heated tobacco products (HTPs) or heat-not-burn (HNB) products. A 2000 internal R J Reynolds document gave the rationale for the pursuit of an acceptable HTP:

Given that no particular agent or group of agents can be definitely assigned the carcinogenic risk associated with cigarettes, the most effective strategy for reducing lung cancer risk in the smoking population is an overall reduction in both the number and concentration of particulate and vapor phase components. This strategy can be achieved by primarily heating, rather than burning, tobacco to form cigarette smoke aerosol.^2^

R J Reynolds first released Premier in 1988,^3^ which was followed by Eclipse, a paper-encased tobacco plug heated by a carbon element.^4^ Independent studies showed that use of Eclipse decreased tobacco cigarette consumption without causing withdrawal symptoms, maintained blood nicotine concentrations and decreased exposure to the carcinogenic tobacco-specific nitrosamine, 4-(methylnitrosamino)−1-(3-pyridyl)−1-butanone, but increased exposure to carbon monoxide (CO).^5–7^ Other HTPs included Philip Morris’ Accord, which was a combination of a handheld device that heated specially constructed cigarettes. One independent study showed that use of Accord suppressed withdrawal symptoms and reduced CO exposure.^8^ Each iteration of HTPs was commercially unsuccessful, and most products were discontinued shortly after their introduction.^9^

Despite repeated failures at producing a commercially viable HTP, tobacco companies continue to research and develop these products. R J Reynolds launched a revamped Eclipse, rebranded as ‘Revo’, in November 2014. Revo was briefly test marketed in Wisconsin but pulled off the market.^10^ Other current HTPs include British American Tobacco’s Glo iFuse, a hybrid of HTP and e-cigarettes. It consists of a heating element, a liquid tank (like e-cigarettes) and a tobacco cavity through which the e-cigarette-like aerosol passes and is infused with tobacco flavour.^11^ Japan Tobacco’s Ploom Tech, which entered the Japanese market in 2016,^12^ consists of a liquid cartridge and a capsule of granulated tobacco leaves that the vapour passes through.

Philip Morris Products S.A., a subsidiary of Philip Morris International, Inc. (PMI), developed IQOS (‘I Quit Ordinary Smoking’) as an HTP.^9 10^ IQOS consists of a tobacco stick (HeatStick) and a battery-powered tobacco heating device.^13^ As of May 2018, IQOS is currently sold in over 37 countries, including Japan, the UK and Canada.^14^ Philip Morris Products S.A. filed a modified risk tobacco product (MRTP) application with the US Food and Drug Administration (FDA) in December 2016^15 16^ to market IQOS in the USA with reduced exposure and reduced risk claims. The FDA’s Tobacco Products Scientific Advisory Committee (TPSAC) reviewed the MRTP application in January 2018. The TPSAC committee approved, in an 8 to 1 vote, PMI’s statement ‘*Scientific studies have shown that switching completely from cigarettes to the IQOS system significantly reduces your body’s exposure to harmful or potentially harmful chemicals [HPHCs*]’ was true.^17^ Of the eight committee members who agreed with PMI’s claim that IQOS significantly reduced exposure to HPHCs, a majority (five of eight) voted that PMI has not ‘*demonstrated that the reductions in exposure are reasonably likely to translate to a measurable and substantial reduction in morbidity and/or mortality*’.^17^

Examination of PMI’s studies, results and interpretation of data to support claims of reduced exposure and risk is critically important before FDA approval in order to protect public health, particularly as PMI’s MRTP application and approval may set the precedent for other MRTP applications of similar products. This paper examines PMI’s reported studies on IQOS aerosol chemistry and human exposure assessment, and we assessed whether they support PMI’s claims of reduced exposure.

## Methods

We examined studies presented in PMI’s MRTP application,^16^ namely those in Module 6.1.1: *Aerosol Chemistry*; Module 6.1.3.1: *Justification of Selection of Biomarkers of Exposure*; and Module 6.1.3.2: *Summary of Biomarkers of Exposure Assessments*. We also reviewed data presented in the document, *Addendum to FDA Briefing Document: January 24–25, 2018*,^18^ which was prepared by FDA’s Center for Tobacco Products for the TPSAC meeting on IQOS held on 24 and 25 January 2018.

To examine the aerosol chemistry of IQOS, mainstream aerosol from IQOS HeatSticks (regular and menthol) and smoke from 3R4F reference cigarettes were generated according to the Health Canada Intense machine-smoking regimen on a Borgwaldt linear smoking machine type LM20X (Borgwaldt KC GmbH, Hamburg, Germany) for most analytes and Burghart rotary smoking machine type RMB 20 (Burghart Tabaktechnik GmbH, Wedel, Germany) for elements.^19^ Methods for chemical analyses have been described previously.^19^

PMI conducted four clinical studies to examine whether human exposure to harmful substances are statistically significantly reduced with IQOS (Module 6.1.3.2).^16^ All studies were randomised, controlled, open-label, three-arm, parallel group, single-centre studies. Studies ZRHR-REXC-03-EU (conducted in Poland) and ZRHR-REXC-04-JP (conducted in Japan) were conducted over 5 days in confinement. Each study included 160 combustible cigarette smokers who were randomly assigned to one of three arms, namely, IQOS with regular HeatSticks, commercially available combustible cigarettes or smoking abstinence. Use of IQOS or combustible cigarettes was from 06:30 to 23:00 and was ad libitum. Studies ZRHM-REXA-07-JP (conducted in Japan) and ZRHM-REXA-08-US (conducted in the USA) were conducted over 3 months, during which 160 participants were randomised to one of three arms in each study, namely, IQOS with menthol HeatSticks, commercially available menthol combustible cigarettes or smoking abstinence. These two studies included 5 days in confinement followed by 85 or 86 days, respectively, in an ambulatory setting. Participants in the IQOS or combustible cigarettes arms used each product ad libitum in confinement (06:30–23:00) and in the ambulatory setting. Compliance with study protocol could not be enforced during the ambulatory phase.

For the two 5-day confinement studies, it was evaluated whether reductions of 50% or more in 24 hours urine concentrations of mercapturic acid metabolites of 1,3-butadiene, acrolein and benzene and carboxyhaemoglobin in blood were observed in smokers assigned to IQOS compared with smokers who continued smoking combustible cigarettes. Levels of selected biomarkers of exposure over the 5-day exposure period were also compared between smokers who switched to IQOS and those who continued smoking and the maximum reduction in biomarker levels in abstinent smokers was assessed. For the two 3-month studies, they examined whether the geometric mean levels of biomarkers of exposure for IQOS (menthol) were lower relative to combustible cigarette (menthol) use. Differences in mercapturic acid metabolites of 1,3-butadiene, acrolein and benzene and carboxyhaemoglobin in blood were tested on day 5 and total 4-(methylnitrosamino)−1-(3-pyridyl)−1-butanol on day 90.

## Results

PMI reported the levels of 58 constituents (which PMI refers to as ‘PMI-58’) in mainstream aerosol generated from IQOS and 3R4F reference cigarettes (Module 6.1.1).^16^ The PMI-58 list includes 40 (43%) out of the 93 harmful or potentially harmful constituents (HPHCs) on FDA’s list of HPHCs.^20^ The PMI-58 list included 18 additional constituents that do not appear on FDA’s list of HPHCs, including water, total particulate matter, pyrene and nitrogen oxides. PMI concluded that the levels of HPHCs on the PMI-58 list were reduced by >92% on a stick basis and >89% on a normalised for nicotine basis for the regular tobacco stick, and >93% on a stick basis and >88% on a normalised for nicotine basis for the mentholated tobacco stick compared to 3R4F reference cigarette (Module 6.1.1, p. 45).^16^

Importantly, the addendum to the briefing document for the 24 and 25 January 2018 TPSAC meeting, prepared by FDA’s Center for Tobacco Products,^18^ presented additional data from PMI studies that showed higher levels of many substances in IQOS emissions compared with 3R4F cigarette smoke (table 1). The addendum consisted of data from Module 3.3.2 and section 6.1.1.3.4 of the MRTP application and appendix A of an amendment to the MRTP application. The addendum reported levels of 113 constituents, including 56 of the 58 constituents on the PMI-58 list (total particulate matter and nicotine-free dry particulate matter were the two exclusions) and 57 constituents that do not appear on the PMI-58 list. Fifty-six of the 57 non-PMI-58 constituents were higher in IQOS emission than in 3R4F smoke (median, 154% higher; range, undefined to 13 650% higher in IQOS aerosol vs 3R4F mainstream smoke); tar was the exception. Twenty-two of the non-PMI-58 constituents were at least 200% higher while seven were at least 1000% higher in IQOS emission compared with 3R4F mainstream smoke (table 1).

**Table 1:** Compounds in mainstream aerosol of Marlboro HeatSticks compared with 3R4F reference cigarette

PMI product	Unit	PMI-58	IQOS HeatStick	3R4F	Change (%) with 3R4F on stick basis
1,2,3-Propanetriol, diacetate (diacetin)	µg/stick	No	1.23	0.381	↑ 223
1,2-Propanediol, 3-chloro	µg/stick	No	9.94	5.93	↑ 68
1,4-Dioxane, 2-ethyl-5-methyl-	µg/stick	No	0.055	0.0004	↑ 13 650
12,14-Labdadiene-7,8-diol, (8a,12E)	µg/stick	No	1.43	0.064	↑ 2134
1 hour-Indene, 2,3-dihydro-1,1,5,6-tetramethyl-	µg/stick	No	0.026	0.014	↑ 86
1-Hydroxy-2-butanone	µg/stick	No	0.947	0.465	↑ 104
1-Hydroxy-2-propanone(1,2-Propenediol)	µg/stick	No	162	96.8	↑ 67
2 (5H)-Furanone	µg/stick	No	5.32	1.99	↑ 167
2,3-Dihydro-5-hydroxy-6-methyl‐4 hour‐pyran‐4‐one	µg/stick	No	0.231	0.135	↑ 71
2,4-Dimethylcyclopent-4-ene-1,3-dione	µg/stick	No	0.333	0.193	↑ 73
2-Cyclopentene-1,4-dione	µg/stick	No	3.8	0.764	↑ 397
2-Formyl-1-methylpyrrole	µg/stick	No	0.128	0.064	↑ 100
2-Furancarboxaldehyde,5-methyl-	µg/stick	No	11.1	2.94	↑ 278
2-Furanmethanol	µg/stick	No	39.2	7	↑ 460
2-Furanmethanol, 5-methyl-	µg/stick	No	0.123	0.029	↑ 324
2 hour-Pyran-2-one,tetrahydro-5-hydroxy	µg/stick	No	4.45	3.11	↑ 43
2-Methylcyclobutane-1,3-dione	µg/stick	No	2.78	0.71	↑ 292
2-Propanone, 1-(acetyloxy)-	µg/stick	No	16.9	8.01	↑ 111
3 (2H)-Furanone, dihydro-2-methyl-	µg/stick	No	0.326	0.119	↑ 174
3-Methylvaleric acid	µg/stick	No	5.1	3.63	↑ 40
4(H)-Pyridine, N-acetyl-	µg/stick	No	0.296	0.112	↑ 164
5-Methylfurfural	µg/stick	No	0.995	0.632	↑ 57
Anhydro linalool oxide	µg/stick	No	0.457	0.291	↑ 57
Benzene, 1,2,3,4-tetramethyl-4-(1-methylethenyl)-	µg/stick	No	0.006	0.005	↑ 20
Benzenemethanol, 4-hydroxy-	µg/stick	No	0.011	0	↑
Benzoic acid, 2,5-dihydroxy-methyl	µg/stick	No	4.55	2.18	↑ 109
Butylated hydroxytoluene	µg/stick	No	0.132	0.007	↑ 1786
Butyrolactone	µg/stick	No	4.08	0.728	↑ 460
Cis-sesquisabinene hydrate	µg/stick	No	0.061	0	↑
Cyclohexane, 1,2-dioxo-	µg/stick	No	0.083	0.046	↑ 80
Cyclohexane-1,2-dione, 3-methyl-	µg/stick	No	0.101	0.073	↑ 38
Eicosane, 2-methyl-	µg/stick	No	0.05	0.014	↑ 257
Ergosterol	µg/stick	No	3.18	1.58	↑ 101
Ethyl 2,4-dioxohexanoate	µg/stick	No	6.73	3.57	↑ 89
Ethyl dodecanoate (ethyl laurate)	µg/stick	No	0.023	0	↑
Ethyl linoleate	µg/stick	No	0.135	0.008	↑ 1588
Ethyl linolenate	µg/stick	No	0.614	0.153	↑ 301
Furfural	µg/stick	No	31.1	25.9	↑ 20
Glycerol	mg/stick	No	5.02	2.08	↑ 141
Glycidol	µg/stick	No	5.71	1.76	↑ 224
Heneicosane, 2-methyl-	µg/stick	No	0.063	0.021	↑ 200
Hexadecanoic acid, ethyl ester	µg/stick	No	0.491	0.008	↑ 6038
Isolinderanolide	µg/stick	No	4.99	1.85	↑ 170
Isoquinoline, 3-methyl	µg/stick	No	6.29	4.99	↑ 26
Labdane-8,15-diol, (13S)	µg/stick	No	0.143	0.015	↑ 853
Lanost-8-en-3-ol, 24-methylene‐, (3beta)	µg/stick	No	6.3	1.61	↑ 291
Maltoxazine	µg/stick	No	0.077	0.038	↑ 103
Methyl furoate	µg/stick	No	0.147	0.029	↑ 407
Phenylacetaldehyde	µg/stick	No	1.41	0.529	↑ 167
p-Menthan-3-ol	µg/stick	No	0.786	0.322	↑ 144
Propylene glycol	µg/stick	No	175	23.7	↑ 638
Pyranone	µg/stick	No	6.54	5.07	↑ 29
Pyranone	µg/stick	No	9.26	5.84	↑ 59
Pyridoxin	µg/stick	No	0.699	0.526	↑ 33
Stearate, ethyl-	µg/stick	No	0.074	0.003	↑ 2367
Tar	mg/stick	No	19.4	25	↓ 22
Trans-4-hydroxymethyl-2-methyl-1,3-dioxolane	µg/stick	No	2.09	0.044	↑ 4650
1,3-Butadiene	µg/stick	Yes	0.21	89.2	↓ 99.8
1-Aminonaphthalene	ng/stick	Yes	0.043	20.9	↓ 99.8
2-Aminonaphthalene	ng/stick	Yes	0.022	17.5	↓ 99.9
3-Aminobiphenyl	ng/stick	Yes	0.007	4.6	↓ 99.8
4-Aminobiphenyl	ng/stick	Yes	0.009	3.21	↓ 99.7
Acetaldehyde	µg/stick	Yes	192	1602	↓ 88
Acetamide	µg/stick	Yes	2.96	13	↓ 77
Acetone	µg/stick	Yes	30.7	653	↓ 95
Acrolein	µg/stick	Yes	8.32	158	↓ 95
Acrylamide	µg/stick	Yes	1.58	4.5	↓ 65
Acrylonitrile	µg/stick	Yes	0.145	21.2	↓ 99.3
Ammonia	µg/stick	Yes	12.2	33.2	↓ 63
Arsenic	ng/stick	Yes	<0.36	<7.49	NA
Benz[a]anthracene	ng/stick	Yes	2.65	28.4	↓ 91
Benzene	µg/stick	Yes	0.45	77.3	↓ 99.4
Benzo[a]pyrene	ng/stick	Yes	0.736	13.3	↓ 94
Butyraldehyde	µg/stick	Yes	20.7	81.3	↓ 74
Cadmium	ng/stick	Yes	<0.28	89.2	↓ >99.7
Carbon monoxide	mg/stick	Yes	0.35	29.4	↓ 99
Catechol	µg/stick	Yes	14	84.1	↓ 83
Chromium	ng/stick	Yes	<11.0	<11.9	NA
Crotonaldehyde	µg/stick	Yes	<3.29	49.3	↓ >93
Dibenz[a,h] anthracene	ng/stick	Yes	<0.124	<0.689	NA
Ethylene oxide	µg/stick	Yes	<0.119	16	↓ >99.3
Formaldehyde	µg/stick	Yes	14.1	79.4	↓ 82
Hydrogen cyanide	µg/stick	Yes	<1.75	329	↓ >99.5
Hydroquinone	µg/stick	Yes	6.55	94.5	↓ 93
Isoprene	µg/stick	Yes	1.51	891	↓ 99.8
Lead	ng/stick	Yes	2.23	31.2	↓ 93
m-Cresol	µg/stick	Yes	0.042	4.24	↓ 99
Mercury	ng/stick	Yes	1.38	3.68	↓ 63
Methyl-ethyl-ketone	µg/stick	Yes	10.1	183	↓ 94
Nickel	ng/stick	Yes	<15.9	<12.9	NA
Nicotine	mg/stick	Yes	1.29	1.74	↓ 26
Nitric oxide	µg/stick	Yes	12.6	484	↓ 97
Nitro benzene	µg/stick	Yes	<0.011	<0.038	NA
Nitrogen oxides	µg/stick	Yes	14.2	538	↓ 97
N-nitrosoanabasine	ng/stick	Yes	2.35	29	↓ 92
N-nitrosoanatabine	ng/stick	Yes	14.7	254	↓ 94
NNK	ng/stick	Yes	7.8	244.7	↓ 97
NNN	ng/stick	Yes	10.1	271	↓ 96
o-Cresol	µg/stick	Yes	0.078	4.81	↓ 98
o-Toluidine	ng/stick	Yes	1.1	96.2	↓ 99
p-Cresol	µg/stick	Yes	0.071	9.6	↓ 99
Phenol	µg/stick	Yes	1.47	15.6	↓ 91
Propionaldehyde	µg/stick	Yes	10.8	109	↓ 90
Propylene oxide	ng/stick	Yes	142.3	896	↓ 84
Pyrene	ng/stick	Yes	8.2	79.2	↓ 90
Pyridine	µg/stick	Yes	6.58	30.9	↓ 79
Quinoline	µg/stick	Yes	<0.011	0.43	↓ >98
Resorcinol	µg/stick	Yes	<0.055	1.72	↓ >97
Selenium	ng/stick	Yes	1.27	<4.42	NA
Styrene	µg/stick	Yes	0.58	13.9	↓ 96
Toluene	µg/stick	Yes	1.42	129	↓ 99
Vinyl chloride	ng/stick	Yes	<0.657	93.4	↓ >99
Water	mg/stick	Yes	30.2	14.7	↑ 105

Notes: presented in table 1 of *Addendum to FDA Briefing Document, January 24-25, 2018*, Meeting of the Tobacco Products Scientific Advisory Committee; sata source: section 3.3.2 and section 6.1.1.3.4 of the Modified Risk Tobacco Product application (MRTPAs) and appendix A of an amendment to the MRTPAs submitted on 8 December 2017. Total particulate matter and nicotine-free dry particulate matter, two constituents on the PMI-58 list were not reported by PMI in this table.

↑, higher in IQOS; ↓, lower in IQOS.

PMI, Philip Morris International; PMI-58, PMI’s list of 58 constituents.

PMI characterised the droplet size distribution of IQOS aerosol by measuring the mass median aerodynamic diameter (MMAD) (the diameter at which 50% of the particles by mass are larger and 50% are smaller) and geometric standard deviation (presented in Module 6.1.1).^16^ The MMAD for the various IQOS products tested (regular and menthol) ranged between 0.54 µm to 0.75 µm and fell within the respirability region, based on the respirability upper threshold defined at 2.5 µm. The range of MMAD for IQOS appears slightly larger than those of e-cigarettes and conventional tobacco cigarettes, which one report showed were about 0.15 µm and 0.17 µm, respectively.^21^

Regarding the human exposure studies, 11 of the 17 HPHCs measured are included in a list of 18 HPHCs that FDA recommends to be measured and reported in users of tobacco products.^20^ PMI assessed systemic exposure to pyrene, which is not included in FDA’s list of HPHCs, as a proxy for exposure to polycyclic aromatic hydrocarbons (PAHs) using 1-hydroxypyrene. PMI did not assess systemic exposure to inorganic compounds, phenols and metals.

Biomarkers of HPHCs measured were statistically significantly lower with IQOS use compared with combustible cigarette use (Module 6.1.3.2).^16^ Reductions of at least 50% in levels of biomarkers of exposure to HPHCs were reported when smokers switched from combustible cigarettes to IQOS during 5 days of confinement; these reductions were sustained during the 85/86 days in ambulatory settings (Module 6.1.3.2, p. 145).

## Discussion

According to FDA’s draft guidance, an MRTP is ‘any tobacco product that is sold or distributed for use to reduce harm or the risk of tobacco-related disease associated with commercially marketed tobacco products’.^22^ FDA may issue an order allowing a product to be marketed as a modified risk product if it is demonstrated that the product: (A) significantly reduces harm and the risk of tobacco-related disease to individual tobacco users; and (B) benefits the health of the population as a whole taking into account both users of tobacco products and persons who do not currently use tobacco products. PMI’s data show that IQOS significantly reduces emissions and exposure to several HPHCs compared with combustible cigarettes. However, PMI’s data also show that IQOS emissions contain higher levels of many other substances compared with combustible cigarettes. The impact of these substances on IQOS toxicity and harm are not known.

Over 7000 distinct substances have been identified in tobacco smoke, many of which are toxic or carcinogenic.^23^ HPHCs in tobacco or tobacco smoke have been proposed by several public health authorities, such as the FDA,^20^ as possible causes of tobacco-related morbidity and mortality. Elimination or reduction of exposure to these HPHCs may potentially reduce health risks, which is the premise of HTP technology. Schaller and colleagues^19^ described five criteria used by PMI to select HPHCs to measure in IQOS aerosol for comparison with 3R4F reference cigarette. Criterion 1 includes smoke constituents determined by International Organization for Standardization (ISO) methods, such as total particulate matter, nicotine and CO. Criterion 2 includes priority toxicants in tobacco smoke selected from the lists issued by regulatory bodies or proposed by cognizant authorities, such as volatile organic compounds like acrylonitrile, 1,3-butadiene and benzene. Criterion 3 includes toxicants for which there is an established biomarker of exposure. Criterion 4 includes toxicants that are predominantly formed below 400°C and that are not included under ‘Criterion 2’, such as acrylamide and acetamide. Criterion 5 includes toxicants that are predominantly formed above 400°C and that are not included under ‘Criterion 1’ and ‘Criterion 2’, such as dibenz[a,h]anthracene and benz[a]anthracene.

PMI’s conclusion that IQOS reduces exposure to HPHCs, which TPSAC agreed with,^17^ is based, in part, on evidence of lower levels of PMI-58 substances in IQOS emissions compared with 3R4F reference cigarette smoke. However, the PMI-58 list is selective (based on PMI’s criteria described before); PMI did not report levels of 53 HPHCs on FDA’s list of 93 HPHCs. Of the 53 FDA HPHCs not measured, 50 are carcinogenic (eg, 2,6-dimethylaniline, benz[j]aceanthrylene, ethylbenzene and furan).^20^ In addition to the PMI-58 substances, PMI measured levels of 57 other substances in IQOS emissions (non-PMI-58 substances). Importantly, 56 of these 57 non-PMI-58 substances were higher in IQOS aerosol compared with 3R4F mainstream cigarette smoke. It appears that IQOS reduces exposure to some toxicants but elevates exposure to other substances.

Given the elevated levels of the non-PMI-58 substances in IQOS aerosol compared with reference cigarette smoke, their inherent toxicities could play a role in the overall harm of IQOS. A number of these substances, including several that were more than 50% higher in IQOS aerosol, belong to chemical classes that are known to have significant toxicity, such as α,β-unsaturated carbonyl compounds (eg, 2-cyclopentene-1,4-dione),^24^ 1,2-dicarbonyl compounds (eg, cyclohexane, 1,2-dioxo-),^25^ furans (eg, 2 (5H)-furanone)^26^ and epoxides (eg, anhydro linalool oxide).^27^ There is limited information on the toxicity of many of the non-PMI-58 substances. We speculate that some of these substances are components of flavour additives in IQOS or thermal degradation compounds. For example, anhydro linalool oxide is listed among flavouring ingredients that are generally regarded as safe (for oral ingestion) by the Flavor and Extract Manufacturers’ Association.^28^2 (5H)-Furanone is a food additive that suppresses appetite and/or food intake and has been shown to induce cellular DNA damage in vitro.^29 30^ 2-Furanmethanol is a flavouring agent with a flavour profile of burnt, caramel or cooked.^31^ 2-Furanmethanol also causes eye, nose, throat and skin irritation and has central nervous system effects.^31^ 2-Cyclopentene-1,4-dione is likely generated from thermal breakdown of sugars.^32^ 1-Hydroxy-2-butanone, a flavouring ingredient found in coffee and coffee products, is also a degradation product of polysaccharides.^33^ Some compounds appear to be contaminants. 3-Chloro-1,2-propanediol (or 1,2-propanediol, 3-chloro), a food contaminant,^34^ has not been shown to be genotoxic in vivo,^35^ but mutagenic effects were observed at high concentrations in vitro experiments.^36^ A 2-year study found increased incidence for the development of tumours in kidney and testis in male rats exposed to 3-chloro-1,2-propanediol.^37^ 1,2,3-Propanetriol, diacetate (diacetin) is a solvent used for decaffeinating coffee.

PMI’s MRTP application fails to address the important question of whether the aerosol generation process for IQOS produces toxic substances not found in the smoke of combustible cigarettes, which could have been answered through non-targeted chemical analysis. Combustible tobacco cigarettes reach about 900°C during a puff and smoulder at about 400°C between puffs.^23^ The burning process, substances emitted and their levels vary at different temperatures.^38^ Distillation, the process during which nicotine and aromas are transferred from tobacco to smoke, occurs below 300°C; pyrolysis occurs at about 300°C–700°C, entails the decomposition of biopolymers, proteins, and other organic materials and generates the majority of substances emitted in smoke; and combustion occurs above 750°C and results in the generation of carbon dioxide, CO and water.^38^ HeatSticks are heated to a maximum of 350°C,^19^ a temperature sufficient to enable pyrolytic decomposition of some organic materials. Formation of toxic volatile organic compounds, including formaldehyde, acetaldehyde and acrolein, via dehydration and oxidation of the humectants, propylene glycol and glycerin, have been reported in e-cigarette aerosols at similar temperatures as IQOS.^39–42^ In addition, flavouring chemicals in e-cigarettes undergo thermal degradation and contribute significantly to levels of toxic aldehydes emitted in e-cigarette aerosol.^43^ Since the constituents of HeatSticks may be different from that of combustible cigarettes, including flavourants and additives, it is plausible that the IQOS aerosol may contain substances not present in tobacco smoke.

A study by Klupinski and colleagues^44^ reported that unique substances, such as ambrox, 3-methylbutanenitrile and 4-methylimidazole, were found in little cigar smoke that were not found in cigarette smoke, indicating that different tobacco products can have different chemical fingerprints and lead to different exposure and toxicological profiles. The study by Klupinski and colleagues describes methodology for ‘non-targeted’ analysis of tobacco smoke aerosol, and the authors suggest that ‘the same approach could also be applied to other samples to characterize constituents associated with tobacco product classes or specific tobacco products of interest’. FDA should recommend that manufacturers of HTPs undertake ‘non-targeted’ analyses (along with targeted analysis), comparing HTP aerosol with smoke from combustible tobacco products to identify potentially toxic chemicals in HTP emissions that may not be present in tobacco smoke.

Although smoking machine studies are appropriate for examining the relative differences in emissions between products, they do not predict use patterns and systemic exposure to toxicants. PMI reported systemic exposure to 17 HPHCs in its human exposure studies. PMI did not assess systemic exposure to any inorganic compounds, phenols and metals, possibly due to the fact that there are no valid biomarkers for some substances or that the time course of the biomarkers may not be optimal for studies of the duration used by PMI. PMI used 1-hydroxypyrene, a metabolite of pyrene (a PAH) as a biomarker of PAHs. Pyrene is not included as an HPHC on FDA’s list. We have previously demonstrated that 1-hydroxypyrene is not a selective measure of tobacco-related PAH exposure and is weakly related to nicotine intake and tobacco-specific nitrosamine exposure.^45^ Instead, we found that monohydroxylated metabolites of fluorene (particularly 1-hydroxyfluorene) and 2-naphthol (a naphthalene metabolite) were more selective of tobacco smoke exposure. In characterising PAH exposure from HNB products, manufacturers should include biomarkers with relatively high selectivity for tobacco.

In conclusion, PMI’s data show that IQOS emissions have significantly lower levels of several HPHCs compared with combustible cigarettes. Furthermore, PMI’s data from human studies show that use of IQOS is associated with significantly lower systemic exposure to some HPHCs compared with smoking combustible cigarettes. These data appear to support PMI’s claim that IQOS is a reduced exposure product. However, PMI’s data also show significantly higher levels of other substances in IQOS emissions compared with combustible cigarette smoke. The impact of these substances on the overall toxicity or harm of IQOS is not known.

**What this paper adds**

- Studies conducted by Philip Morris International, Inc. (PMI) show that IQOS emissions contain lower levels of many harmful or potentially harmful constituents (HPHCs) compared with combustible tobacco smoke.
- PMI’s studies show that use of IQOS results in significantly lower systemic exposure to several HPHCs compared with combustible cigarette smoking.
- PMI’s own data also show that IQOS emissions contain many other substances, some of which are potentially toxic, at higher levels than in combustible cigarette smoke.
